# Proteolysis and Rheological Properties of Cream Cheese Made with a Plant-Derived Coagulant from *Solanum elaeagnifolium*

**DOI:** 10.3390/foods8020044

**Published:** 2019-01-30

**Authors:** Néstor Gutiérrez-Méndez, Alejandro Balderrama-Carmona, Socorro E. García-Sandoval, Pamela Ramírez-Vigil, Martha Y. Leal-Ramos, Antonio García-Triana

**Affiliations:** Facultad de Ciencias Químicas, Universidad Autónoma de Chihuahua, Chihuahua 31125, Mexico; alexbcarmona@gmail.com (A.B.-C.); eliizabeth.gs@gmail.com (S.E.G.-S.); pamelarvigil@hotmail.com (P.R.-V.); yarelyleal@hotmail.com (M.Y.L.-R.); triana.antonio@gmail.com (A.G.-T.)

**Keywords:** plant-derived coagulant, *Solanum elaeagnifolium*, acid-rennet-curd cheese, cream cheese, proteolysis, texture

## Abstract

Cream cheese is a fresh acid-curd cheese with pH values of 4.5–4.8. Some manufacturers add a small volume of rennet at the beginning of milk fermentation to improve the texture of the cream cheese. However, there is no information about the effect that proteases other than chymosin-like plant-derived proteases may have on cream cheese manufacture. This work aimed to describe some proteolytic features of the protease extracted from fruits of *Solanum elaeagnifolium* Cavanilles and to assess the impact that this plant coagulant has on the viscoelastic properties of cream cheeses. Results showed that caseins were not hydrolyzed extensively by this plant-derived coagulant. In consequence, the ratio of milk clotting units (*U*) to proteolytic activity (*U-Tyr*) was higher (1184.4 *U*/*U-Tyr*) than reported for other plant proteases. The plant coagulant modified neither yield nor composition of cream cheeses, but viscoelastic properties did. Cream cheeses made with chymosin had a loss tangent value (*tan* δ = 0.257) higher than observed in cheeses made with 0.8 mL of plant-derived coagulant per liter (*tan* δ = 0.239). It is likely that casein fragments released by the plant-derived coagulant improve the interaction of protein during the formation of acid curds, leading to an increase in the viscoelastic properties of cream cheese.

## 1. Introduction

Cream cheese is a fresh acid-curd cheese fermented with lactic acid bacteria at pH values of ~4.5–4.8 [1,2]. During acidification of milk, colloidal calcium phosphate (CCP) in the casein micelles are solubilized [3]. If the pH decreases below 5.0, most of CCP dissolves and casein micelles become dissociated [2]. When the pH in the milk reaches a value ~4.6, the negative surface charge on casein micelles will be reduced enough to induce the formation of a gel structure [4]. The firmness of acid-milk gels can be improved by either a preheat treatment of milk [2,5] or by a small addition of rennet [3]. Heat treatment of milk above 69 °C denatures the whey proteins (β-lactoglobulin (β-lg) and α-lactalbumin (α-la)) which associate with caseins [2]. Aggregation of β-lg with κ-casein reduces the net repulsive charge among caseins, enhancing the protein–protein interactions and thus the gel firmness [6]. On the other hand, addition of a small amount of rennet (chymosin) at the beginning of fermentation induces a coarser network of proteins that enhances the firmness of milk gels [3]. Chymosin hydrolyzes caseins (mostly κ-casein) generating *para*-κ-casein and a glycomacropeptide. However, it is not clear enough how the release of these casein fragments improves the interactions among proteins during formation of acid-milk gels [7]. Furthermore, there is no information about the impact of proteases other than chymosin in the formation of acid-enzymatic gels. 

For centuries, the coagulant used for cheese making was obtained from calf stomach (calf rennet). Nowadays, calf rennet alone cannot cover the current demand for cheese-making coagulating enzymes. Recombinant *Bos taurus* chymosin and microbial-origin coagulants are alternative milk clotting enzymes widely used in the dairy industry nowadays [8]. Plant-derived coagulants have also been studied as possible substitutes for calf rennet, though in reality only a few are used for commercial cheese-making [9]. Most plant proteases are not suitable milk clotting agents because of their excessive proteolytic nature or their low ratio of milk clotting activity to proteolytic activity [8,9]. However, some plant-derived proteases are successfully used in the manufacture of commercial cheeses, for instance, plant-derived coagulants obtained from *Cynara cardunculus* L. and certain *Solanum* plants. The aspartic proteases from *Cynara* sp. are used to produce a large variety of cheeses in the Mediterranean, West Africa, and southern European countries [9]. Meanwhile, plant-derived coagulants from *Solanum dubium* Fresen and *Solanum elaeagnofolium* Cavanilles are used in the manufacture of commercial cheeses in Sudan [10] and Mexico [11]. 

**Figure 1:**
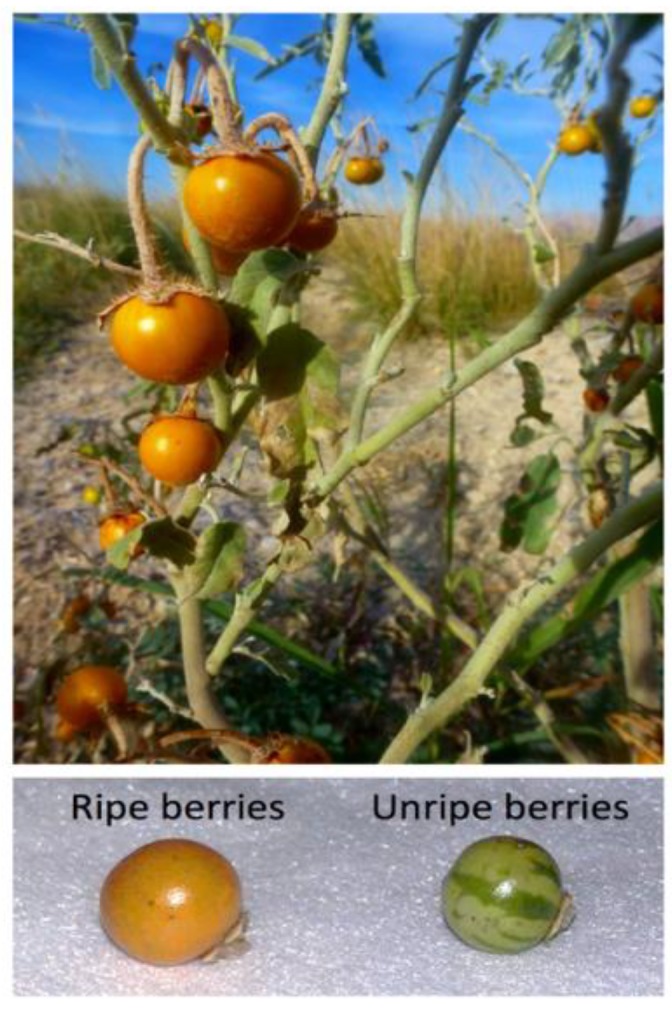
Photograph of the fruits of *Solanum elaeagnifolium*.

In the northeast of Mexico, the fruits of *S. elaeagnifolium* (Figure 1) have been used for decades in the manufacture of asadero cheese, a pasta-filata-type cheese [12]. The use of this plant for asadero cheese manufacture was first described between 1916 [13] and 1924 [14]. Unfortunately, the protease from *S. elaeagnifolium* is lesser-known and not as well studied as proteases from *Cynara* sp. or *S. dubium.* Some of the most interesting features of protease from *S. elaeagnifolium* is its high ratio of milk clotting activity to proteolytic activity [11]. Additionally, this plant-derived coagulant can induce the formation of stable milk-gels and curds with a soft texture, particularly at mild-acidic conditions [11,12]. In consequence, the protease from fruits of *S. elaeagnifolium* could be used in the manufacture of acid-enzymatic curd cheeses with a soft texture. This work aimed to describe some proteolytic features of the protease extracted from ripening fruits of *S. elaeagnifolium*, as well as to assess the usage of this plant-derived coagulant in the manufacture of cream cheese. 

## 2. Materials and Methods

### 2.1. Plant Material

Ripe yellow fruits were collected from native wild plants of *S. elaeagnifolium* (Figure 1) growing in Chihuahua City and the municipality of Julimes, Mexico. Berries were gathered during the late fall to ensure they were sun-dried. The dry fruits were milled with a mini-mill and sieved with a mesh of 0.5 mm (3383-L19, Thomas Wiley Fisher, Swedesboro, NJ, USA). The powder was stored in hermetically sealed glass vessels at −20 °C. Compositional analysis of the fruits powder was carried out with the AOAC methods 942.05 (ash), 948.22 (fat), 960.52 (protein), 934.01 (moisture) and 991.43 (total, soluble, and insoluble fiber) [15].

### 2.2. Extraction of Proteases from Fruit Powder

Plant-derived proteases were extracted by mixing the fruit powder with sodium acetate buffer (50 mM, 5%NaCl, pH 5) at a 1:10 ratio (*w*/*v*). The mixture was incubated for 12 h at 4 °C under magnetic stirring and then centrifuged twice at 3200× *g* for 20 min at 4 °C. The supernatant was recovered and filtered first with paper Whatman no. 1 (GE Healthcare, Buckinghamshire, UK) and then through polyethersulfone filters of 0.45 μm (Whatman, GE Healthcare, Buckinghamshire, UK). The content of protein in the plant-derived coagulant was quantified by the Bradford method [16]. The filtered extracts were stored at −20 °C in hermetically sealed plastic tubes.

### 2.3. Proteolytic Properties of the Plant-Derived Coagulant

The effect that the plant-derived coagulant had on caseins (α_S1_, α_S2_, β, and κ) and whey proteins (β-lg, and α-la) were assessed by polyacrylamide gel electrophoresis in the presence of sodium dodecyl sulfate (SDS-PAGE). For this analysis, a solution of casein and a solution of whey proteins were prepared at 1% (*w*/*v*). The reaction of proteolysis was carried out by mixing 130 μL of the protein solution with 25 μL of plant coagulant. After 10 min of incubation at 37 °C, the reaction was stopped by boiling the solution for 15 min. The mixture was cooled to room temperature and loaded in the polyacrylamide gel. The conditions of the SDS-PAGE were according to the methodology of Chavez-Garay et al. [11]. The photographs of gels were digitized and analyzed with the software ImageJ (National Institutes of Health, Bethesda, MD, USA). 

The proteolytic activity of the plant-derived coagulant and chymosin was measured in triplicate with a modified Lowry method [17]. Briefly, 130 μL of casein solution (0.66% *w*/*v*) were added with 25 μL of plant coagulant and incubated at 37 °C for 10 min. The proteolytic reaction was ended by adding 130 μL of trichloroacetic acid (100 mM). The hydrolyzed solution of casein was centrifuged at 3000× *g* for 15 min at 10 °C to sediment the non-hydrolized caseins. A volume of 250 μL was taken from the supernatant and placed into an empty tube. To this tube was added 625 μL of Na_2_CO_3_ (500 mM) and 125 μL of Folin-Ciocalteu solution (0.2 N), and it was incubated at 37 °C for 30 min. The supernatant was read at 760 nm in a microplate reader (Biotek, Elx808, Winooski, VT, USA). Data were interpreted with the calibration curve made with standards of tyrosine.

### 2.4. Capacity of the Plant-Derived Coagulant to Clot Milk

Milk clotting activity was estimated in both the plant coagulant and chymosin (Chy-Max, Chr Hanse, Horsholm, Denmark) according to the methodology described by Chávez-Garay et al. [11]. Results were expressed as Soxhlet units of coagulation (*U*) using Equation (1). Soxhlet units or milk clotting units (*U*) represents the volume of milk that can be clotted by one volume unit of coagulant in 40 min at 35 °C [8]. Thus, one volume of chymosin can clot a larger amount of milk than one volume of the plant-derived coagulant. In this equation, *t_clotting_* was the timespan required to clot milk, whereas *V_milk_* and *V_coagulant_* represent the milliliters of milk and coagulant used in the enzymatic reaction. Additionally, the specific milk clotting activity (*specific_MCA_*) was calculated with Equation (2). [protein] is the concentration of protein expressed as mg/mL of plant coagulant, and *mL_coagulant_* is the volume of coagulant used. The *specific_MCA_* was expressed as *U*/mg of protein coagulant. Milk clotting activities of the plant coagulant and the chymosin were measured five times.

(1) $$U=\frac{V_{milk}}{V_{coagulant}}\times \frac{40\;min}{t_{clotting}}$$

(2) $$specific_{\;MCA}=\frac{U/mL_{coagulnt}}{\left(protein\right)}$$

### 2.5. Manufacture of Cream Cheese

The milk was heated to 80 °C for 10 min, cooled to 38 °C and poured into glass vessels (Pyrex, Corning, Tewksbury, MA, USA) in portions of 500 mL. Four portions of milk were used for each treatment; consequently, each treatment was analyzed four times. To the milk was added 400 μL of CaCl_2_ (6.6 M) and inoculated (~1% *w*/*v*) with a freeze-dried starter culture of *L. lactis* spp. (Choozit, Danisco, Niebüll, Germany). The plant-derived coagulant was added at three different volumes in the milk portions. The amounts added were 200, 400 and 800 μL, being equivalent to 0.4, 0.8, and 1.6 μg of protein per milliliter of milk. In the case of cheese made with chymosin (control), milk portions has 10 μL of the coagulant added (0.022 μg of protein/mL of milk). Chymosin from *Aspergillus niger* var. Awamori, kindly donated by Chr Hansen (Chy-Max, Horsholm, Denmark) was used. After the addition of coagulants, the vessels were incubated at 36 °C, reaching a pH of 4.8 (after ~7 h). Curds were then transferred into cloth bags and centrifuged at 1700× *g* for 30 min at 10 °C to remove the whey fraction (Thermo IEC, Needham Heights, MA, USA). The cream cheeses were packed in polyethylene bags and stored at 10 °C. The yields of cream cheeses were calculated with the formula described by Chávez-Garay et al. [11]. All cheeses were subjected to proximate analysis using AOAC methods [15].

### 2.6. Evaluation of Proteolysis in Cream Cheeses

Proteolysis in cream cheeses was evaluated by SDS-PAGE. The protein fractions in the cream cheeses were obtained by mixing 250 mg of cheese with Tris-HCl buffer pH 8 (165 mM Tris, 1 mM EDTA, 70 mM SDS). The aqueous fractions were loaded onto a polyacrylamide gel (stacking 5%, separating 12%) with enough volume to obtain a concentration of ~45 μg of protein. SDS-PAGE conditions and analysis were as described elsewhere in the text.

### 2.7. Spreadability of Cream Cheese

Spreadability of the cream cheeses was measured using a texture analyzer TA.XTplus (Stable Micro System, Godalming, UK) with a cell load of 50 kg. Before the analysis, a conical base was filled with cream cheese and stored at 10 °C for two hours. Meanwhile, a plastic (perspex) cone with a 45° angle was fitted in the arm of the texturometer. Then, both the cone and the conical base were aligned vertically in the texturometer. The upper cone penetrated the lower base at a speed of 1 mm/s until it reached a distance of 54 mm, which was 2 mm above the bottom of the lower conical base. The force required to obtain the maximum penetration depth of 2 mm was taken to be the spreadability of the cream cheese [1,18].

### 2.8. Viscoelastic Properties of Cream Cheese

Viscoelastic properties of cream cheeses were measured with a rheometer AR-2000 (TA Instrument, New Castle, DE, USA) and using dynamic small-amplitude oscillatory tests. Cheese samples were placed between an aluminum (0° angle) plate of 40 mm in diameter and the rheometer platform (gap = 2 mm). Before the stress sweep analysis, the linear viscoelastic region was established by strain sweep analysis at different frequencies. Stress sweeps were carried out with the following test parameters: oscillated stress from 1 to 1000 Pa, logarithmic mode, frequency 1.5 Hz, and temperature of 5 °C. The storage modulus (G′), loss modulus (G″), complex viscosity (η*), and phase angle (δ) were obtained from the linear viscoelastic region observed during stress sweeps [18]. Results were also expressed in terms of the loss tangent (tan δ), which represents the dimensionless ratio G″/G′. 

## 3. Results and Discussion

### 3.1. Gross Composition of S. elaeagnifolium Fruits

The composition of dried-yellow berries from *S. elaeagnifolium* was similar to reported by Chávez-Garay et al. [11], excepting for the fat content (Table 1). The source of fat in the powder of dried fruits was likely the seeds, whereas the fruit peels contributed to the high content of insoluble fiber. Proteins in the dried fruits are probably distributed between the seeds and the fruit peels, since milk clotting activity has been detected in both [11]. The amount of protein was ~14 g/100 g of fruit powder (Table 1). Considering the content of protein and the number of fruits produced by a single plant (40 to 60) [19], these solanaceous fruits could be an available source of proteases.

### 3.2. Proteolytic Properties of the Plant-Derived Coagulant

**Figure 2:**
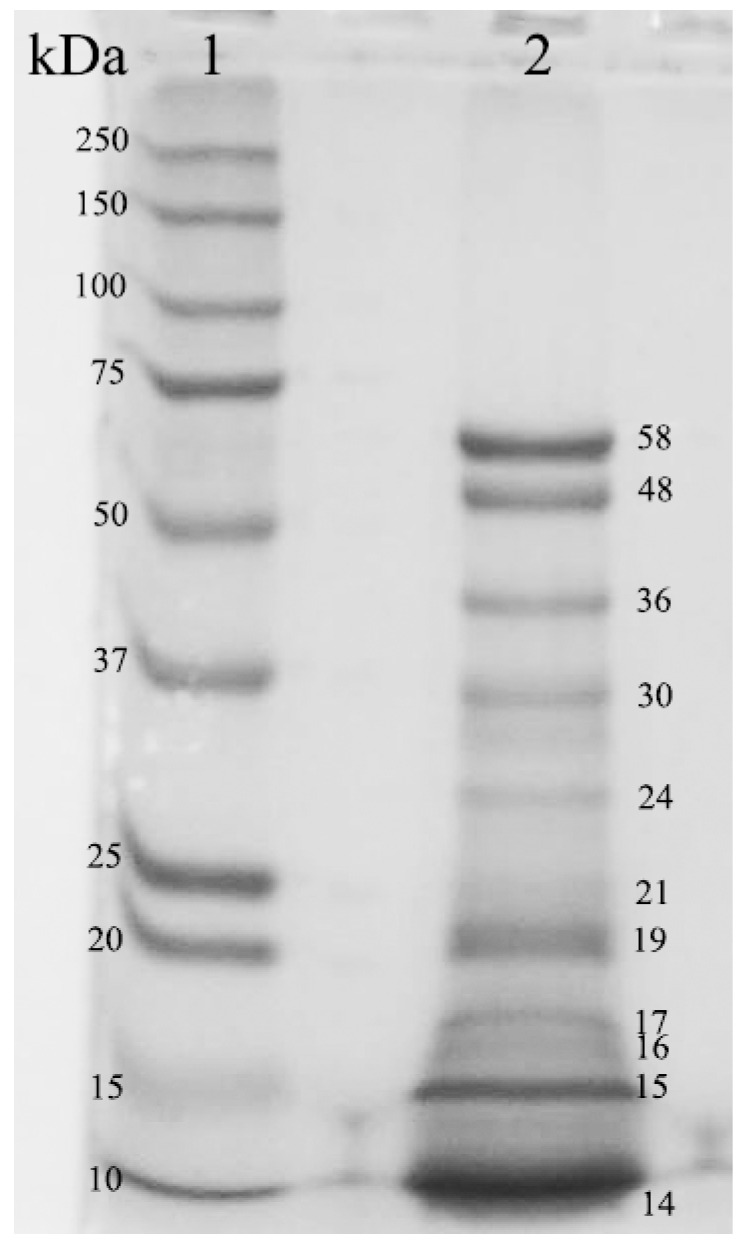
Polyacrylamide gel electrophoresis of the plant coagulant obtained from the yellow berries of *S. elaeagnifolium*. Lane 1: molecular weight marker, lane 2: plant coagulant.

The plant coagulant had a proteolytic activity of 1.52 ± 0.097 *U-Tyr*/mL and specific proteolytic activity of 0.78 ± 0.045 *U-Tyr*/mg of protein. In comparison, chymosin had a low proteolytic activity (0.29 ± 0.13 *U-Tyr*/mL) and a high specific activity (2.51 ± 0.08 *U-Tyr*/mg of protein). The augmented specific activity of chymosin was due to the high purity of the enzyme. In counterpart, the plant-derived coagulant had a mixture of diverse proteins in addition to proteases. Figure 2 shows the number of proteins in the plant coagulant and their corresponding molecular weights. Protease in fruits of *S. elaeagnifolium* has a molecular weight (MW) of ~58 kDa [11], that is, the first band in the second lane of Figure 2. A milk clotting enzyme with similar MW (66 kDa) has been reported in the fruits of *Solanum dubium*. This protease (Dubium) was identified as a chymotrypsin-like serine protease by assessment with protease inhibitors [20].

**Figure 3:**
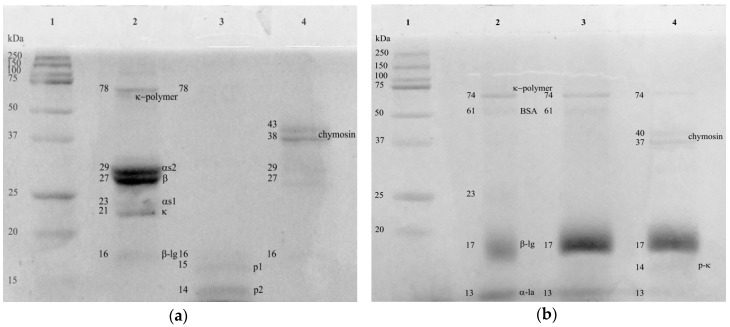
Casein (**a**) and whey protein (**b**) solutions at 1% before (lane 2 in both images) and after (lane 3 in both images) being treated with either the plant coagulant or chymosin (lane 4 in both images) for 10 min at 37 °C. Lane 1 (both images): molecular weight marker. P1 and P2 peptides derived from the hydrolysis of caseins with the plant coagulant.

According to the SDS-PAGE analysis, protease from the fruits of *S. elaeagnifolium* was able to hydrolyze caseins (Figure 3a) but not whey proteins (Figure 3b). The plant-derived coagulant hydrolyzed the four types of caseins: α_s1_-, α_s2_-, β- and κ-casein. From casein hydrolysis, two peptides (p1 and p2) with MWs of 15 and 14.5 kDa were observed (Figure 3a). However, peptides smaller than 10 kDa (not detected by SDS-PAGE analysis) could have been generated by the plant coagulant. Chymosin hydrolyzed κ-casein and partially α_S1_-, α_S2_-, and β-casein (Figure 3a). It is well known that chymosin has a limited proteolytic activity on α_S1_-, α_S2_-, and β-casein but is particularly active on κ-casein [21,22]. The specific hydrolysis of chymosin on κ-casein (at Phe_105_-Met_106_) releases a hydrophilic glycoprotein (glycomacropeptide) and leaves a positively charged para-κ-casein attached to casein micelles. Consequently, the repulsive electric force among casein micelles decreases, as well as the steric repulsion which favors the clot of milk [22]. On the other hand, the plant-derived coagulant did not hydrolyze the whey proteins (β-lg and α-la) or produce a slight hydrolysis, which could not be detected by SDS-PAGE analysis. In contrast, it was confirmed in the same polyacrylamide gel (Figure 3b) that chymosin hydrolyzed α-la, as reported by other authors [22]. Unlike caseins, whey proteins are globular proteins with a relatively high hydrophobicity and compactly folded peptide chains [23]. Therefore, whey proteins are less susceptible to hydrolysis by proteases than caseins.

### 3.3. Milk Clotting Properties of the Plant-Derived Coagulant

*S. elaeagnifolium* fruit showed the ability to clot milk (978.36 ± 88.3 *U*/mL), yet not as good as chymosin from *Aspergillus niger* (2285.70 ± 102.3 *U*/mL). The milk clotting activity observed in the plant coagulant was similar to that reported for the fruits of *S. dubium* (880 *U*/mL), a closely related plant [10]. When the specific-milk clotting activities (specific _MCA_) were calculated, the disparity between chymosin and plant coagulant was even more evident (20,779.09 and 923.85 *U*/mg of protein). This increased difference was anticipated, since the purity and quantity of enzyme varied between both coagulants. For instance, 1 mL of chymosin solution contained 0.113 mg of protein or enzyme. In contrast, 1 mL of the plant-derived coagulant had 1.0597 mg of protein, but only a minor fraction of this protein corresponded to proteinases. The ratios of milk clotting activity to proteolytic activity (MCA/PA) were 8278.5 and 1184.4 *U*/*U-Tyr* for chymosin and the plant-derived coagulant, respectively. 

Different authors have stated that proteases from plants have a lower capacity to clot milk than rennin or chymosin [9]. The specific hydrolysis of chymosin on κ-casein decreases the zeta potential of casein micelles (~20 mV) by half and reduces the steric repulsion among micelles. These changes greatly favor the aggregation of casein micelles and thus the clot of milk [21,22]. The high milk clotting capacity of chymosin, having a relatively low proteolytic activity, gives this enzyme an elevated MCA/PA ratio. The higher the MCA/PA ratio the better the use of coagulant for cheese making, because the clotting time is short and the volume of coagulant needed is low [21,24]. Proteases in the plant-derived coagulant hydrolyzed not only the κ-casein but also the α_S1_-, α_S2_-, and β-casein (as described elsewhere in the text). Even though the hydrolysis of caseins by the plant coagulant was unspecific, it was enough to induce clotting of milk. The MCA/PA ratio of the plant coagulant was lower than chymosin but higher than reported for other plant coagulants like Neriifolin S, papain, trypsin, ficin and religiosin (433, 367, 3.6, 393, and 387 *U*/OD 660 nm) [24,25]. Therefore, proteases from the berries of *S. elaeagnifolium* are suitable for cheese-making. Indeed, this plant coagulant has been used for decades in the manufacture of artisanal asadero cheese in the northwest of Mexico [26].

### 3.4. Composition of Cream Cheese Made with the Plant-Derived Coagulant

Excepting fat content, the composition of cream cheeses was similar to that reported for commercial brands of either Neufchatel or full-fat cream cheese [1]. The fat content in our cream cheeses ranged from 14–15% (Table 2), whereas commercial Neufchatel and full-fat cream cheese have 20–25% and 32–35% of fat, respectively [1]. As previously described, cream cheeses were manufactured using three different concentrations of plant coagulant (0.4, 0.8, and 1.6 μg protein/mL milk) and the control cheeses were made with chymosin. One primary concern for using plant proteases for cheese-making is the loss of fat and proteins in the cheese whey, which reduces the cheese yield. This problem arises from the excessive and unspecific proteolysis that some plant coagulants have on caseins, leading to the formation of weak milk-gel structures [21]. Proteases from the fruits of *S. elaeagnifolium* modified neither fat, lactose, nor ash content in cream cheeses at any plant coagulant concentration (Table 2). The maximal concentration of plant-derived coagulant brought about a cream cheese slightly wetter, with lower protein content than control cheese (Table 2). However, the yields of cream cheeses manufactured with the plant-derived coagulant were only marginally reduced (statistically not significant), regardless of the concentration of plant coagulant used (Table 2).

### 3.5. Proteolysis in Cream Cheeses

**Figure 4:**
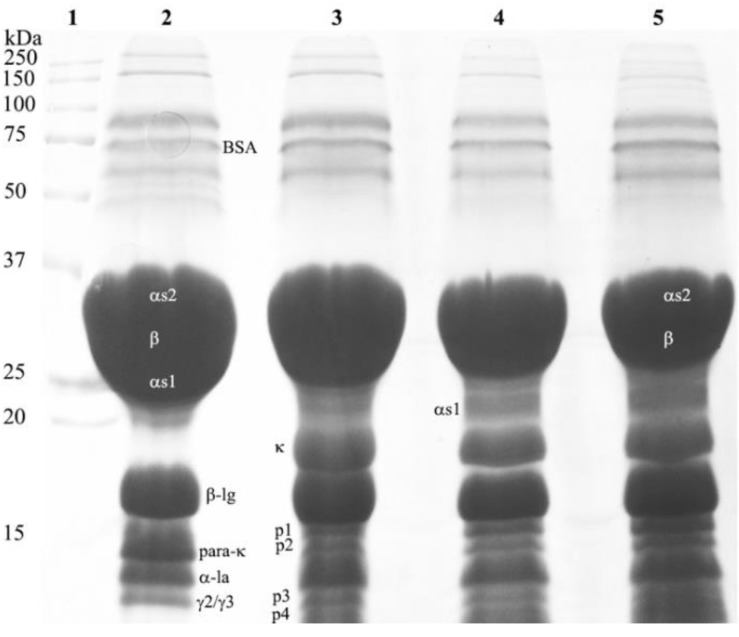
SDS-PAGE of aqueous extracts obtained from cream cheeses. Lane 1: molecular weight marker; lane 2: cheese made with chymosin; lane 3: cheese clotted with a plant coagulant (0.042 mg of protein/100 mL of milk); lane 4: cheese clotted with a plant coagulant (0.0846 mg of protein/100 mL of milk); lane 5: cheese clotted with a plant coagulant (0.1694 mg of protein/100 mL of milk).

**Figure 5:**
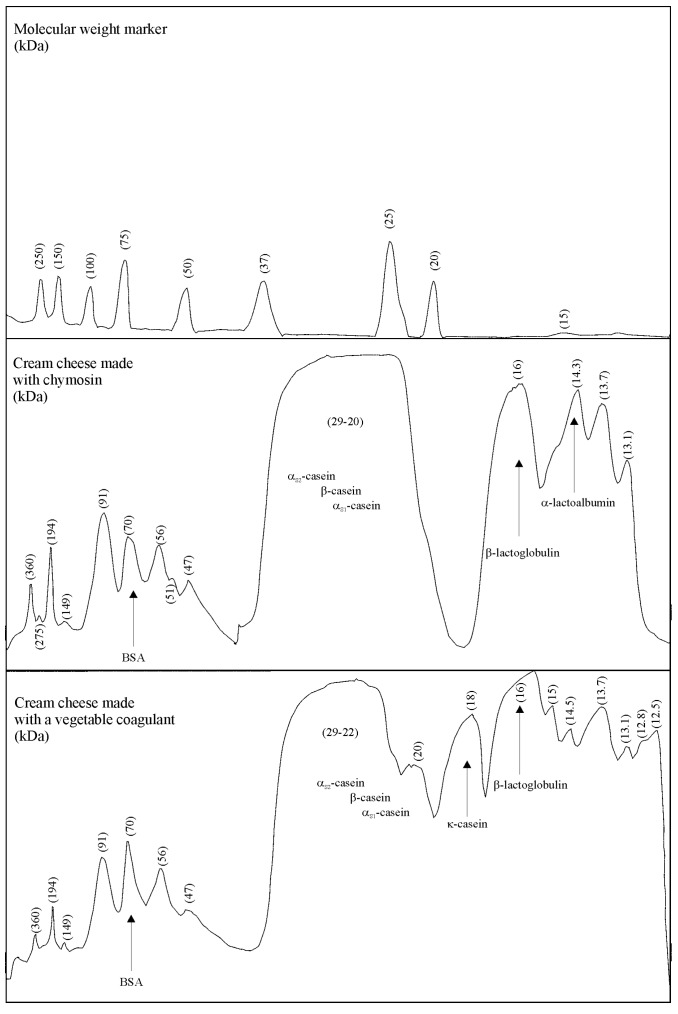
Densitometric analysis of SDS-PAGE with samples of cream cheese made with chymosin and cream cheese made with the plant coagulant obtained from the berries of *S. elaeagnifolium*.

The gel electrophoresis analysis unveiled that all caseins (αs_1_-, αs_2_-, β-, and κ-casein) were partially hydrolyzed in cream cheeses manufactured with the plant-derived coagulant. Conversely, chymosin extensively degraded κ-casein in cream cheeses but scarcely hydrolyzed other type of caseins (Figure 4 and Figure 5). The incremental addition of plant-derived coagulant enhanced the proteolysis of caseins in cream cheeses, particularly the breakdown of αs_1_-casein (Figure 5). SDS-PAGE analysis of the cheeses also confirmed the low proteolytic activity that plant protease has on caseins. As previously described, the plant-derived coagulant had lower specific proteolytic activity on caseins (0.78 ± 0.045 *U-Tyr*/mg of protein) than chymosin (2.51 ± 0.08 *U-Tyr*/mg of protein). It is worthwhile highlighting these results, because most plant proteases exhibit high levels of proteolytic activity on caseins, which leads to the formation of multiple small peptides. Formation of such peptides can impair texture and produce a bitter flavor in the cheese [27,28]. Only four peptides were detected in the cheese made with the plant coagulant: p1 = 15 kDa, p2 =14.5 kDa, p3 = 12.8 kDa, p4 = 12.5 kDa (Figure 4). Bitter peptides are mostly hydrophobic and arise from the breakdown of α_s1_-, and β-casein [29]. Some authors have suggested that bitter peptides produced by milk coagulants (such as calf rennet, microbial, and plant proteases like cardosin) have a MW of 0.15 to 14 kDa [29]. Other authors have reported that bitter peptides consist of 2 to 23 amino acid residues with MW ranging from 0.5 to 3 kDa [30].

### 3.6. Rheological Properties of Cream Cheese

Cream cheeses clotted with the fruits of *S. elaeagnifolium* had spreadability values comparable to those coagulated with chymosin (the control cheese). However, the viscoelastic properties of the cream cheeses varied significantly depending on the coagulant and volume of coagulant used (Table 3). For instance, cream cheese clotted with a small quantity of plant coagulant (0.4 mL of coagulant/L milk) had lower values of η*, G′, and G′′ than control chymosin cheese. Doubling the volume of plant coagulant (0.8 mL of coagulant/L milk) resulted in cream cheese with viscoelastic properties similar to the control cheese. However, the opposite effect was observed with a further volume increase of plant-derived coagulant (1.6 mL of coagulant/L milk), which notably decreased the viscoelastic properties of cream cheese.

Addition of rennet during gel formation in acid-curd cheeses (like cream cheese) accelerate milk clotting and induce a coarser protein network that enhances the gel firmness [3,7]. It is, however, necessary to add an adequate amount of coagulant in the milk (critical rennet concentration, CRC) to synchronize the acid and rennet clotting. Otherwise, a mild or adverse effect could be observed by a low or excessive addition of rennet [3]. It was noted that cream cheeses with 0.8 mL of plant coagulant per liter of milk added had the lowest value of tan δ—0.239 ± 0.01 (or highest G′ value). It is likely that such plant coagulant concentration not only hydrolyzed caseins but also did so at the proper rate. Thus, cross-linking between proteins (caseins and whey proteins) and peptides could have been favored. Nevertheless, a lower concentration of plant coagulant (0.4 mL of coagulant/L milk) produced insufficient hydrolysis of caseins (Figure 4) which increased the value of tan δ (0.246 ± 0.05). Similarly, a concentration of plant coagulant higher than 0.8 mL of coagulant/L milk led to extensive hydrolysis of caseins (Figure 4), raising tan δ to 0.276 ± 0.05. The increment of tan δ in a combined rennet and bacterial fermentation indicates an imbalance of negative charges on the micelles, which weakens the milk-gel structure [7,31]. 

Cream cheeses made with chymosin had a tan δ value (0.257 ± 0.015) higher than that observed in cheeses made with 0.8 mL of plant-derived coagulant per liter (tan δ = 0.239 ± 0.01). As previously described, the plant protease degraded all the caseins (α_S1_-, α_S2_-, β-, and κ-casein) slightly, releasing four peptides (p1, p2, p3, and p4). In contrast, chymosin extensively broke up κ-casein with a concomitant formation of para-κ-casein and a glycomacropeptide (Figure 3 and Figure 5). It is possible that peptides released by the plant-derived coagulant enhanced the interactions among caseins in a better way than casein fractions released by chymosin.

## 4. Conclusions

*S. elaeagnifolium* is considered a weed by farmers. However, the dry fruits of this plant contain a milk clotting protease at high concentration. This plant-derived protease had a lower ratio of milk clotting activity to proteolytic activity (*MCA/PA*) than chymosin, but a *MCA/PA* higher than reported for other plant proteases. With reference to cream cheese, it yields and composition that did not change with the use of the plant-derived coagulant. Only the maximal concentration of plant-derived coagulant brought about a cream cheese slightly wetter and with lower protein content than a control cheese. SDS-PAGE analysis revealed that proteases from *S. elaeagnifolium* hydrolyzed all caseins, but only partially. From this hydrolysis, four peptides ranging from 12.5 to 15 kDa were observed. The unspecific hydrolysis produced by the plant protease modified the viscoelastic properties of the cream cheeses produced. We found that the critical enzyme concentration (CRC) for this plant coagulant is 0.8 mL of plant coagulant per liter of milk (0.8 μg of total protein per mm of milk). According to the results obtained, this concentration enhances the interaction between proteins and peptides, leading to an increase in the viscoelastic properties of cream cheese.

## Figures and Tables

**Table 1:** Compositional analysis of the dried yellow berries of *Solanum elaeagnifolium* used in this study.

Component	g/100 g of Dried Fruits
Moisture	5.8 ± 0.1
Fat	5.0 ± 0.1
Ash	5.8 ± 0.08
Protein	13.8 ± 0.3
Total fiber	56.8 ± 2
Soluble fiber	2.9 ± 0.9
Insoluble fiber	53.8 ± 1.1
Carbohydrates ^1^	12.8

^1^ Estimated by difference.

**Table 2:** Composition and yield of cream cheeses made with chymosin and a plant coagulant obtained from the ripe fruits of *S. elaeagnifolium.*

(PROTEASE Added) μg of Protein/mL of Milk	(0.022)	(0.4)	(0.8)	(1.6)
Component	Control ^1^		Cheese made with plant coagulant	
Moisture (g/100 g)	59.7 ± 8.8 ^a^	59.5 ± 6.5 ^a^	58.5 ± 4.4 ^a^	61.9 ± 2.8 ^a^
Fat (g/100 g)	14.4 ± 0.6 ^a^	14.4 ± 0.7 ^a^	14.9 ± 0.1 ^a^	14.3 ± 0.7 ^a^
Protein (g/100 g)	6.0 ± 0.4 ^a^	5.1 ± 0.2 ^ab^	5.3 ± 0.8 ^ab^	4.7 ± 0.6 ^b^
Ash (g/100 g)	1.1 ± 0.1 ^a^	1.0 ± 0.05 ^a^	1.0 ± 0.1 ^a^	0.8 ± 0.2 ^a^
Carbohydrates ^2^ (g/100 g)	18.8 ± 2.0 ^a^	20.1 ± 1.8 ^a^	20.3 ± 1.9 ^a^	18.3 ± 2.1 ^a^
Yield (%)	16.1 ± 1.21 ^a^	14.9 ± 0.74 ^a^	14.0 ± 2.4 ^a^	15.0 ± 2.1 ^a^

^1^ Cheese made with chymosin from *Aspergillus niger* var. *awamori* (Chr Hansen). ^2^ Calculated by difference. SE = standard error. ^a, b^ Means followed by different letters in the same row are statistically different (ANOVA, Tukey-Kramer test, *p* < 0.05). Data represent the average of four replicates (*n*).

**Table 3:** Rheological properties of cream cheeses made with chymosin and a plant coagulant obtained from the ripe berries of *S. elaeagnifolium.*

(Protease Added) μg of Protein/mL of Milk	(0.022)	(0.4)	(0.8)	(1.6)
Item	Control ^1^		Cheese made with plant coagulant	
Spreadability ^2^ (N)	151.3 ± 27.8 ^a^	117.7 ± 8.1 ^a^	151.6 ± 23.3 ^a^	101.3 ± 25.8 ^a^
η* (kPa×s)	20.4 ± 7.8 ^ab^	10.5± 3.9 ^bc^	29.2 ± 11.2^a^	4.4 ± 0.77 ^c^
G′ (kPa)	124.3 ± 28.9 ^b^	64.1 ± 35.5 ^bc^	178.6 ± 18.9 ^a^	26.8 ± 4.8 ^c^
G″ (kPa)	32.1± 13.9 ^a^	15.8 ± 4.4 ^b^	42.8 ± 13.3 ^a^	7.4 ± 2.9 ^b^

^1^ Cheese made with chymosin from *Aspergillus niger* var. *awamori* (Chr Hansen). ^2^ Force required to spread the cheese between two surfaces. SE = standard error. η* = complex viscosity. G′ = storage modulus. G″ = loss modulus. ^a, b^ Means followed by different letters in the same row are statistically different (ANOVA, Tukey-Kramer test, *p* < 0.05). Data represent the average of four replicates (*n*).
